# Acute Liver and Renal Failure: A Rare Adverse Effect Exclusive to Intravenous form of Amiodarone

**DOI:** 10.1155/2016/5232804

**Published:** 2016-09-08

**Authors:** Robin Paudel, Prerna Dogra, Saurav Suman, Saurav Acharya, Jyoti Matta

**Affiliations:** ^1^Department of Internal Medicine, University of Kentucky, Lexington, KY, USA; ^2^Department of Internal Medicine, Jersey City Medical Center, Jersey City, NJ, USA

## Abstract

Amiodarone is an antiarrhythmic drug which is highly effective against a wide spectrum of ventricular tachyarrhythmias making it irreplaceable in certain group of patients. We report an unusual case of acute liver and renal failure within 24 hours of initiation of intravenous (IV) amiodarone which resolved after stopping the medication. The mechanism of acute liver and renal toxicity is not clearly known but is believed to be secondary to amiodarone induced (relative) hypotension, idiosyncratic reaction to the drug, and toxicity of the vector that carries the medication, polysorbate-80. In this case review, we discuss the hyperacute drug toxicity caused by IV amiodarone being a distinctly different entity compared to the adverse effects shown by oral amiodarone and support the suggestion that oral amiodarone can be safely administered even in patients who manifest acute hepatitis with the IV form.

## 1. Introduction

Amiodarone is a class III antiarrhythmic drug highly effective against a wide spectrum of ventricular tachyarrhythmias. We report an unusual case of acute liver and renal failure within 24 hours of initiation of intravenous (IV) amiodarone. Despite wide popularity and efficacy of amiodarone in the treatment of various kinds of cardiac arrhythmias, little is understood about the mechanisms by which IV amiodarone can lead to acute liver and renal failure. As a result, most of the physicians end up withholding this medication altogether. In this case review, we discuss the hyperacute drug toxicity caused by IV amiodarone being a distinctly different entitycompared to the adverse effects shown by oral amiodarone and support the suggestion that oral amiodarone can be safely administered even in patients who manifest acute hepatitis with the IV form.

## 2. Case Summary

65-year-old Caucasian male with past medical history of Coronary Artery Disease (CAD) status post Coronary Artery Bypass Graft Surgery (CABG), Ischemic Cardiomyopathy status post Automatic Implantable Cardioverter Defibrillator (AICD), hypertension, and dyslipidemia presented to the emergency room (ER) with complaints of multiple AICD shocks. In the ER, he was hemodynamically stable and in no acute distress. His physical examination was unremarkable and had no signs of decompensated heart failure. His initial set of labs at presentation that included Complete Blood Count (CBC), Basic Metabolic Panel (BMP), and Liver Function Test (LFT) were within normal limits. Echocardiogram showed severely dilated left ventricle with ejection fraction of 23%. Patient was loaded with amiodarone 150 mg IV followed by amiodarone drip (1 mg/min for first 6 hours and then 0.5 mg/min for next 18 hours). Patient received total of 1050 mg. Patient was continued on his home medications (Docusate 100 mg three times a day as needed, aspirin 81 mg daily, clopidogrel 75 mg daily, famotidine 20 mg two times a day, escitalopram 10 mg daily, valsartan 80 mg daily, metoprolol succinate 100 mg daily, ezetimibe 10 mg daily, and simvastatin 80 mg daily). In addition, he also received enoxaparin 40 mg daily and zolpidem 5 mg daily.

**Figure 1:**
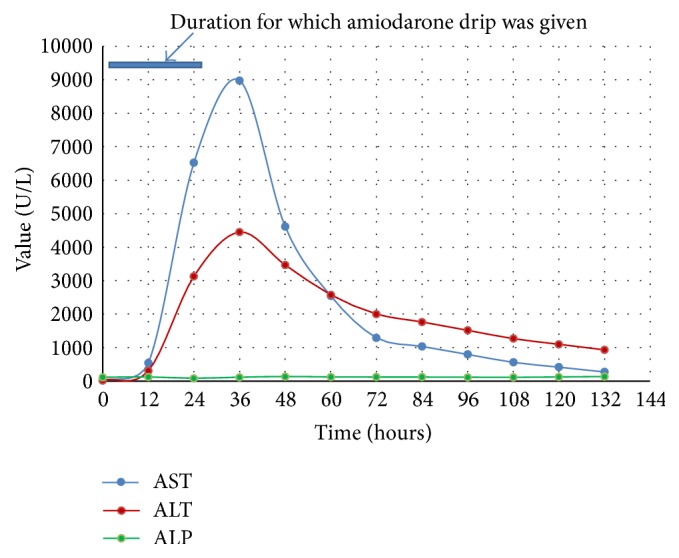
Elevation of liver enzymes after IV amiodarone.

**Figure 2:**
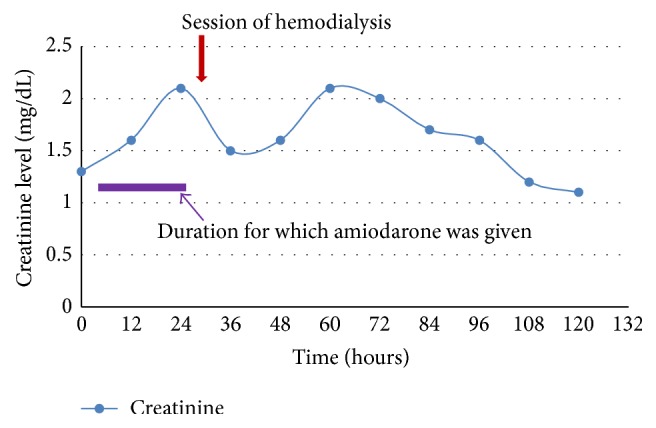
Worsening renal function after IV amiodarone.

His BP at presentation was 140/88(MAP 105) which dropped to the lowest, 90/60(MAP 70), over the initial 5 to 6 hours of starting amiodarone and remained in that range for 2 to 3 hours. It then improved to MAP of 70–75 but remained lower than his baseline MAP around 100. During the last one-third duration of the amiodarone drip, patient had another period of hypotension with the lowest systolic BP of 86 and diastolic BP of 49 with MAP ranging from 61 to 75 lasting for a period of 3 hours. All other causes of hypotension including sepsis, hypovolemia, and cardiac arrhythmia were ruled out. His blood pressure began to improve 2 hours after stopping amiodarone. However, labs showed dramatic changes. Lab works done one day after starting amiodarone showed marked increase in WBC, potassium, LFTs (Figure 1), and creatinine (Figure 2). Patient became oliguric and hyperkalemic requiring emergent hemodialysis. Ultrasound of the right upper quadrant of the abdomen showed normal liver architecture, workup for viral hepatitis was negative, and sepsis was ruled out.

Patient was monitored in the hospital over 5 days for downward trend of LFTs and creatinine and was discharged home with arrangements for further follow-up plans. After one month, oral amiodarone was started which was well tolerated by the patient with follow-up lab works done at 6 months showing normal values.

## 3. Discussion

Amiodarone is an iodinated-benzofuran class III antiarrhythmic drug which is highly effective against a wide spectrum of ventricular tachyarrhythmias. Amiodarone is notoriously known to cause various adverse effects including but not limited to liver toxicity, hypo- or hyperthyroidism, QT prolongation, Atrioventricular (AV) block, severe hypotension, Acute Respiratory Distress Syndrome (ARDS), cardiogenic shock, pulmonary fibrosis, and visual disturbance. Amiodarone, being extremely lipophilic, enters the tissues easily and accumulates in the cells including hepatocytes. With a half-life of around 6 months, most of the common serious adverse effects are mainly seen on long term use, oral route being the mode of administration. Amiodarone is initially given at a higher dosage orally or IV termed as the loading dose followed by a daily maintenance dose orally. While most of the known toxic effects of amiodarone is seen on prolonged oral use, most acute adverse effects are observed with the intravenous use of the medication. For unknown reasons, few of the rare side effects that include acute liver failure, cardiac arrest, ARDS, renal injury, and hypotension are almost exclusively seen with the intravenous administration of amiodarone and not with oral loading or maintenance dosing of amiodarone [1–4]. We have presented one such rare case of acute liver and renal failure secondary to intravenous amiodarone administration. The diagnosis of amiodarone toxicity was made based on a thorough drug history excluding any other hepatotoxic agents and other causes of hepatitis, complete physical examination, laboratory investigation, and the temporal relationship with amiodarone administration and rapid improvement in liver/renal function after stopping amiodarone.

Hepatotoxicity caused by* oral* amiodarone is well documented with around 15–20% patients on amiodarone shown to have elevation in levels of transaminases [2, 5–7]. It can lead to 1.5- to 4-fold increase in the level of amino transferases (AST/ALT) while there will be minimal increase in Gamma Glutamyl Transferase (GGT) and generally no change in the level of bilirubin and alkaline phosphatase [5]. Despite elevation of the liver enzymes, clinically significant liver injury warranting discontinuation of amiodarone remains low at around 1%.

On the other hand,* intravenous (IV)* amiodarone toxicity can raise AST/ALT up to 100–200-fold (*AST of 8981 and ALT of 4450 in our case*) within a day of infusion which then reverses quickly after discontinuation of amiodarone except for a few reported cases of fatal hepatotoxicity [8, 9]. There have been case reports where patients who manifested acute hepatotoxicity when started on IV amiodarone tolerated the oral amiodarone quite well and manifested acute toxicity when IV form was reintroduced [10, 11]. While the toxicity from chronic oral amiodarone is believed to be due to direct damage to the lipid bilayers and the disturbance of lysosomal and mitochondrial function, the acute toxicity due to IV amiodarone is believed to be due to different mechanisms like hypersensitivity reaction, relative hypotension causing ischemic liver injury [10, 12, 13], toxicity of the vehicle (polysorbate-80) [11, 14, 15], and idiosyncratic toxicity. Centrilobular necrosis and collapse with minimal inflammation and no appreciable fat as seen in these patients with acute liver toxicity with intravenous amiodarone toxicity suggest ischemic cause of liver injury. Ischemic liver injury is understood to be caused by relative hypotension in the setting of congestive hepatopathy secondary to underlying congestive heart failure which most of the patients needing amiodarone would probably have. Our patient did not have increase in eosinophils count in the blood which points against hypersensitivity reaction. The fact that the mean arterial pressure (MAP) dropped on average by 30–40 mm Hg compared to his baseline favors the etiology being ischemic. His BP remained low throughout the course of amiodarone administration only to improve after stopping amiodarone.

There is also some literature suggestive of the vehicle polysorbate-80 as the cause behind the acute toxicity seen in intravenous amiodarone administration [11, 14, 15]. Polysorbate-80 or polyoxyethylene-sorbitan-20 monooleate (C64H124O26) is a vehicle used to make the stable solution form of amiodarone for intravenous use. Polysorbate-80 has been shown to have similar hepatotoxic and nephrotoxic effect, popularly known as* E-ferol syndrome* [14–19] seen in the infants who get intravenous form of vitamin E which contains polysorbate-80 and polysorbate-20.

Not much has been studied about the kidney injury seen in amiodarone. Fogoros et al. [5] reported that about 8.8% of patients on oral amiodarone had an elevation of creatinine with a mean increase of 0.81 mg/dL with one among them requiring discontinuation of the medication. The mechanism behind renal injury due to amiodarone is not clearly understood with renal failure secondary to rhabdomyolysis and cardiorenal syndrome due to drop in blood pressure being the proposed mechanisms. In our patient, cardiorenal syndrome is the most likely mechanism.

Although acute toxic effect due to intravenous amiodarone is rare, but occurred once, it creates a true dilemma in the physician's mind regarding its future use in the patient. While failure to recognize acute toxicity of IV amiodarone can be detrimental, unnecessary avoidance of amiodarone can preclude a patient from getting a potentially lifesaving medication. Our case, for instance, presented with 16 episodes of sustained ventricular tachycardia, 10 of them being terminated by AICD firing and the need for effective antiarrhythmic cannot be overemphasized.

Etiology behind acute toxicity associated with IV amiodarone is still not completely understood. However, irrespective of the etiology, taking into consideration that the acute toxicity of IV amiodarone is a distinctly different entity from the chronic toxicities of oral amiodarone, we support the recommendation not to withhold the potentially lifesaving oral amiodarone after the patient has an acute toxicity due to IV amiodarone. Oral amiodarone can be safely started once the liver enzymes normalize [10, 11, 15], as done in our patient, although further monitoring of the liver enzymes is recommended once the patient is started on oral amiodarone.

## Additional Points

*Objective.* We report a case of acute liver and renal failure induced by intravenous amiodarone and review of similar cases.
